# Volumetric Properties and Stiffness Modulus of Asphalt Concrete Mixtures Made with Selected Quarry Fillers: Experimental Investigation and Machine Learning Prediction

**DOI:** 10.3390/ma16031017

**Published:** 2023-01-22

**Authors:** Fabio Rondinella, Fabiola Daneluz, Pavla Vacková, Jan Valentin, Nicola Baldo

**Affiliations:** 1 Polytechnic Department of Engineering and Architecture (DPIA), University of Udine, Via del Cotonificio 114, 33100 Udine, Italy; 2 Faculty of Civil Engineering, Czech Technical University in Prague, Thákurova 7, 166 29 Prague, Czech Republic

**Keywords:** asphalt mixtures, alternative fillers, XRF analyses, artificial intelligence, machine learning, decision tree, CatBoost

## Abstract

In recent years, the attention of many researchers in the field of pavement engineering has focused on the search for alternative fillers that could replace Portland cement and traditional limestone in the production of asphalt mixtures. In addition, from a Czech perspective, there was the need to determine the quality of asphalt mixtures prepared with selected fillers provided by different local quarries and suppliers. This paper discusses an experimental investigation and a machine learning modeling carried out by a decision tree CatBoost approach, based on experimentally determined volumetric and mechanical properties of fine-grained asphalt concretes prepared with selected quarry fillers used as an alternative to traditional limestone and Portland cement. Air voids content and stiffness modulus at 15 °C were predicted on the basis of seven input variables, including bulk density, a categorical variable distinguishing the aggregates’ quarry of origin, and five main filler-oxide contents determined by means of X-ray fluorescence spectrometry. All mixtures were prepared by fixing the filler content at 10% by mass, with a bitumen content of 6% (PG 160/220), and with roughly the same grading curve. Model predictive performance was evaluated in terms of six different evaluation metrics with Pearson correlation and coefficient of determination always higher than 0.96 and 0.92, respectively. Based on the results obtained, this study could represent a forward feasibility study on the mathematical prediction of the asphalt mixtures’ mechanical behavior on the basis of its filler mineralogical composition.

## 1. Introduction

A flexible road pavement is mainly made of aggregates, bituminous binder, and mineral filler, and its mechanical behavior is deeply affected by the physical–chemical characteristics of these three basic components and by their mutual interaction. During their service life, pavements have to withstand traffic and climate loads and must be carefully designed both in terms of mixture and layer thicknesses. Otherwise, common failure phenomena such as permanent deformation, low-temperature cracking, fatigue, and stripping could occur, reducing pavement service life and increasing rehabilitation costs [1,2].

Experimental methods are currently performed to characterize the mechanical behavior of construction materials [3,4,5], pavement asphalt mixtures included [6,7,8,9,10], even though expensive laboratory equipment is usually required. Despite the experience of researchers and technicians, any modification to the mixture’s composition always involves additional laboratory tests leading to an increase in the cost and time required to fully design the mixture.

A mathematical or numerical model would overcome this issue by allowing each parameter to be individually adjusted and by providing accurate predictions of the mixture’s mechanical response. For this reason, many researchers have developed and proposed predictive equations and models that relied on the mechanics of materials and referred to advanced constitutive modeling methods. The mechanical behavior of asphalt mixtures has thus been described and elaborated by means of rational constitutive laws [11,12,13] that were later implemented in finite element [14,15,16] and discrete element software [17,18,19].

Although such mathematical models provide an in-depth physical understanding of asphalt mixtures’ mechanical response, statistical approaches and machine learning methods are recently gaining wide approval in the scientific community. Unlike constitutive equations, they are independent of the problems of physical nature but can successfully achieve fast and reliable results [20,21,22,23]. However, in direct comparison, machine learning-based methods such as artificial neural networks (ANNs) and decision trees (DTs) have been proven to produce more accurate predictions than corresponding statistical approaches [24,25,26,27,28,29,30]. An ANN is a soft-computing technique inspired by the functioning principles of the human nervous system that processes information by means of basic computational units (neurons) and their interconnection. Although neural networks can successfully understand and model even highly nonlinear phenomena producing very accurate predictions [31,32,33,34,35,36,37], the difficulties related to the best hyperparameters’ identification and the lack of sufficient interpretability [38] could make them not preferable. Conversely, decision tree-based models solve regression and/or classification problems by means of simple and easily interpretable decision rules [39], returning a performance that is competitive with that of neural networks [40,41].

In recent years, many interesting decision tree-based predictive models were realized, capable of analyzing and evaluating the behavior of asphalt mixtures. Benhood and Daneshvar implemented the M5P model tree algorithm to successfully predict the dynamic modulus $\left|E^{*}\right|$ of asphalt concretes [42]. The same predictive task was also proficiently accomplished by Ali et al. implementing an eXtreme Gradient Boosting-based methodology [43]. Hosseini et al. were able to predict the viscoelastic behavior of modified bitumen in terms of complex shear modulus ($G^{*}$) and phase angle (δ) by means of decision trees and ensemble regression methods [44]. Recently, Liu et al. improved the mix design process by predicting alligator cracking and longitudinal cracking from asphalt mixture properties by means of Gradient Boosting, eXtreme Gradient Boosting, and extra-trees algorithms [45].

The main purpose of this study was to develop and implement an innovative decision tree-based methodology to accurately predict the volumetric and stiffness properties of asphalt concrete mixtures from the mineralogical composition of the fillers used. To achieve this goal, 126 specimens prepared with different alternative quarry fillers were analyzed, keeping the filler content at 10%, fixing bitumen type (paving grade bitumen 160/220) according to EN 1744-4, Annex A, and binder content (6%) and with roughly the same grading curve. For all the experimentally designed and assessed mix variants, the bulk density, voids content, stiffness at 15 °C according to EN 12697-26, annex C and Marshall test at 60 °C according to EN 12697-34 were determined. X-ray fluorescence (XRF) spectrometry analyses were also performed to determine the five main filler-oxide contents.

A categorical boosting (CatBoost) approach was implemented to identify a reliable correlation between two predicted outputs, namely the air voids’ content (AV) and the stiffness modulus at 15 °C (IT-CY), and seven input variables including the bulk density, five oxide contents, and a categorical variable distinguishing the aggregates’ quarry of origin.

## 2. Materials and Methods

For the assessment of the effect of different fillers, derived mainly from quarry dust, on the characteristics of an asphalt mix (deformation behavior, durability, and adhesion of bitumen to aggregate), representatives of aggregates from the Zbečno, Brant, and Chlum quarries were selected as they represent the different types of rocks available in the Czech Republic and are regularly used for the production of asphalt mixes. This selection includes aggregates showing a different adhesion to bituminous binders.

With respect to Zbečno quarry, the parent rock is igneous. Petrographically, it is a spilite with plagioclase strips (andesite) and pyroxene isometric grains. Quartz, calcite, chlorite, or pumpellyite are abundantly contained in secondary veins. Up to 3 mm of feldspar outgrowths (spilite porphyrites) can be found in some spilites. Zbečno aggregates usually show a good adhesion with bitumen.

Granite porphyry can be considered the key mineral of the Brant quarry rock. Although its surface is porous due to weathering, it is also hydrophilic and consequently more susceptible to loss of adhesion with the asphalt binder.

The aggregate from the Chlum quarry in the northern region of the country can be classified as an acid rock type (phonolite). Feldspars are not detectable macroscopically; biotite can be found in small quantities. This rock-produced aggregate is typically more hydrophilic, showing poor adhesion of asphalt to the aggregate. Therefore, in the case of this aggregate, the mix design usually requires proper adhesion promoters. The alternative solution—if possible—is to try to avoid this type of aggregate in asphalt mix design.

In addition, this study used a soft paving-grade bitumen 160/220 with 187 dmm of penetration, and 38 °C of softening point. This binder-type is requested by the test procedure described in Annex A of EN 1744-4, which was chosen as an alternative method to assess the suitability of the filler in the asphalt mix (the procedure is generally not well-known in Central European latitudes, but its use has a very long history according to the literature). The exact grading-curve composition is defined in Annex A, where 25% 5/8 mm, 25% 2/5 mm, 40% 0.125/2 mm, and 10% of the filler must be represented. This atypically defined grading curve requires, in particular for the standard 0/2 mm fraction, the removal of particles <0.125 mm, which are completely replaced by filler. The closest type of an asphalt mix according to EN 13108-1 would be an ACsurf 8, eventually, according to EN 13108-2, some of the BBTM 8 mix types. The asphalt content is optimized to achieve for the reference mix a voids’ content uniformity of 5.5 ± 0.5% vol. This has to be defined for each type of aggregate and the base asphalt mix with the reference limestone filler (in this study, the Velké Hydčice quarry) was used. The bitumen content determined for the reference mix was used for all alternatives considered where a different type of filler was used to replace the limestone meal. As alternative fillers used to replace the traditional limestone filler, several variants of quarry dust or backhouse fillers from asphalt mix production representing different quarries or in two cases asphalt mixing plants were chosen. Quarry dust (QD) came from the quarries of Plešovice, Litice, Chrtníky, and Chornice. The backhouse filler (BF) was collected from the Brant (Froněk) and Kladno (PKB) asphalt plants. More detailed data on the fillers used and their typical properties important for use in asphalt mixtures can be found in a recently published paper [46].

### 2.1. Spectrometry Test

This analysis was based on the generally established classification whereby a sample containing more than 65% SiO_2_ is considered to be an acidic origin rock and it is usually hydrophilic. Conversely, a higher content of CaO indicates that the material can be considered hydrophobic. An ARL QUANT’X EDXRF spectrometer (Thermo Scientific, Waltham, MA, USA) equipped with an Rh X-ray tube and a Si(Li) detector crystal was used. XRF spectrometry data were collected and analyzed using UniQuant ED 6.32 software (Thermo Scientific, USA). Using this equipment, the relative accuracy varies between 0.5% and 5.0% depending on the amount and concentration of the analytes.

### 2.2. CatBoost Model

To understand whether it was feasible to predict the mechanical and volumetric properties of an asphalt mixture on the basis of its compositional variables and filler oxide contents, a decision tree-based machine learning technique called Categorical Boosting (CatBoost) was implemented. It improves the well-known gradient-boosting decision tree by significantly enhancing its data-fitting capabilities [47]. By combining the use of balanced decision trees and an algorithm known as ordered boosting [48], CatBoost has proven to outperform other modern gradient-boosting decision tree-based techniques [49] such as LightGBM [50] and XGBoost [51]. Finally, a unique processing flow is performed for categorical features [52]. The formal analytical functioning of CatBoost is accurately described by Prokhorenkova et al. [48].

Multiple combinations of the model’s hyperparameters were investigated to identify the one that would optimize its performance. A short summary of the comprehensive grid search has been provided in Table 1. Fine-tuned hyperparameters are represented by the number of iterations, the maximum depth of the trees, and the learning rate.

The k-fold cross-validation technique was also introduced to properly assess the model’s generalization capabilities according to Equation (1), and an overfitting detector was implemented to prevent the occurrence of overfitting phenomena. k and overfitting detector-values were set equal to 5 and 20, respectively, in accordance with relevant literature [53,54].

(1) $$\mathrm{Loss}\;\mathrm{function}\;\left(k\right)=\frac{1}{k}\sum_{i=1}^{k}{\mathrm{Loss}\;\mathrm{function}}_{\;i}$$

The identification of the best model was based on the lowest loss function-value. MultiRMSE was chosen as loss function since two parameters were simultaneously predicted, and its value was analytically determined as:

(2) $$\mathrm{MultiRMSE}=\sqrt{\frac{1}{N}\sum_{i=1}^{N}\sum_{d=1}^{D}{\left(y_{T_{i,d}}-y_{P_{i,d}}\right)}^{2}}$$

where $y_{T_{i}}$ was the i-th true value; $y_{P_{i}}$ was the i-th CatBoost prediction; D was the number of output variables, and N was the number of observations included in the test vector.

Before the dataset was processed by the model, laboratory results were normalized in accordance with Equation (3). For each variable, all observations are mapped to the range [0, +1] so that the lower and the upper limits are representative of the minimum and the maximum values, respectively. This is a common practice in machine learning since models have proven to be more effective when different data are scaled to the same range [55].

(3) $$x_{\mathrm{norm}}=\frac{x\;-{\;x}_{\min}}{x_{\max}-{\;x}_{\min}}$$

To fully characterize the performance of the CatBoost model, six different evaluation metrics were implemented and evaluated:

The mean absolute error (MAE):

(4) $$\mathrm{MAE}=\frac{1}{N}\sum_{i=1}^{N}\left|y_{T_{i}}-y_{P_{i}}\right|$$

The mean absolute percentage error (MAPE):

(5) $$\mathrm{MAPE}=\frac{1}{N}\sum_{i=1}^{N}\left|\frac{y_{T_{i}}-y_{P_{i}}}{y_{T_{i}}}\right|\times 100$$

The mean squared error (MSE):

(6) $$\mathrm{MSE}=\frac{1}{N}\sum_{i=1}^{N}{\left(y_{T_{i}}-y_{P_{i}}\right)}^{2}$$

The root mean squared error (RMSE):

(7) $$\mathrm{RMSE}=\sqrt{\frac{1}{N}\sum_{i=1}^{N}{\left(y_{T_{i}}-y_{P_{i}}\right)}^{2}}$$

The Pearson correlation coefficient (R):

(8) $$R=\frac{1}{N-1}\sum_{i=1}^{N}\left(\frac{y_{T_{i}}-\mu_{y_{T_{i}}}}{\sigma_{y_{T_{i}}}}\right)\left(\frac{y_{P_{i}}-\mu_{y_{P_{i}}}}{\sigma_{y_{P_{i}}}}\right)$$

The coefficient of determination (R^2^):

(9) $$R^{2}=1-\frac{\sum_{i=1}^{N}{\left(y_{T_{i}}-y_{P_{i}}\right)}^{2}}{\sum_{i=1}^{N}{\left(y_{T_{i}}-\mu_{y_{T_{i}}}\right)}^{2}}$$

For each predicted variable, the terms µ and σ represent the mean value and standard deviation, respectively. The outlined methodology was implemented in Python 3.8.5.

## 3. Results and Discussion

### 3.1. Laboratory Results

**Figure 1:**
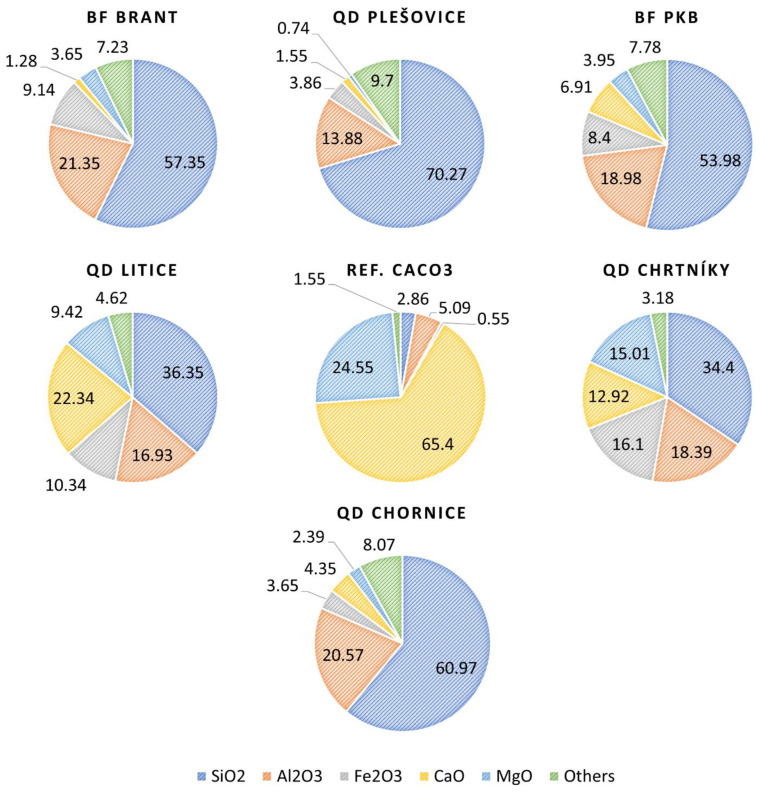
Quarry fillers’ oxide contents determined by means of XRF spectrometry (m/m%).

The used and tested alternative filler samples were in terms of XRF spectroscopy automatically evaluated in a helium atmosphere at 25 °C over the entire spectral range measurable by the spectrometer. Figure 1 shows the XRF results summarizing the most significant oxides found in the fracture dust or reversible filler samples, later used for machine learning and modeling tasks. The results are divided into three series of asphalt mixes. Each series represents one type of used aggregate (mineral type) with 7 variations of fillers. As stated earlier, the asphalt mixtures of each series were produced under the same laboratory conditions using same compaction energy.

From the results presented in Table 2a, alternative fillers can have a significant effect on the air voids content of the asphalt mix. With respect to the reference mixture prepared with limestone filler, an air voids content equal to 5.33% vol. was shown (highest among the three reference mixtures), with the bitumen content in this case equal to 6.3% hm. Only in the case of replacing the traditional filler with an alternative material in the form of Plešovice quarry dust similar voids content value was reached. The Brant and PKB backhouse fillers exhibited significantly higher voids, which would likely have resulted in a requirement for a slight increase in the bitumen content to achieve the same voids as the asphalt mix with limestone filler option. On the other hand, the quarry dust from Litice and Chornice resulted in a lower voids content. These results demonstrate very well that it is not only the content of the dosed filler that is crucial, but also its physical and geometrical characteristics that will affect the volumetric properties of the asphalt mixture.

From the results shown in Table 2b, the alternative fillers influence the voids content and densities in this series of asphalt mixtures. The reference mix containing limestone filler had a voids content of 5.01% vol. (lower than e.g., in the case of Zbečno aggregate). The dosed bitumen content was slightly higher and reached 6.4% hm. In this case, the claim of a possible influence of the tested fillers on the volumetric properties is, according to the results, valid for the quarry dusts from Plešovice, Litice and Chornice, with the most significant influence found for the first three of these alternative fillers.

From the results presented in Table 2c, the selected alternative fillers can affect voids content value. This may be related to the shape of the particles, their size, as well as the surface of the filler particles, which is described e.g., in the study presented by Antunes et al. [56]. In this research work, it was shown that there is an influence of the geometrical and physical properties of the fillers on the bitumen-filler interaction and the peeling resistance of the bituminous binder. The reference mix containing limestone filler had a voids content of 5.18% vol. According to the results obtained, the claim about the potential influence of the tested fillers on the asphalt mix volumetric properties is especially true for the variant with PKB backhouse filler and Chornice quarry dust.

- With respect to literature studies which have considered asphalt mixtures with composition roughly similar to those of the current research, Tušar et al. [57] have discussed the low temperatures resistance of asphalt mixtures AC 8, which presented bulk densities, air voids, Marshall flow values within the range 2.404–2.484 g/cm^3^, 1.8–8.0%, 4.1–6.0 mm, respectively, depending on the specific composition. Hribar et al. [58] have also investigated the low temperatures properties of asphalt mixtures AC 8 surf, characterized by bulk densities and air voids values within the range 2.411–2.483 g/cm^3^ and 2.3–8.4%, respectively, depending on the bitumen content. Therefore, both the cited literature papers have outlined experimental results pretty similar to those of the current study.
- According to the observed mechanical and deformation characteristics presented in Table 2a, the asphalt mixture with the QD Plešovice filler showed the highest stiffness modulus, while the PKB backhouse filler and the reference mixture with limestone filler demonstrated the lowest stiffness. The remaining variants had similar stiffness values, which were close to the stiffness exhibited by the mix with PKB backhouse filler or limestone. The stiffness results for the QD Plešovice variant and the variant with PKB backhouse filler corresponded well with the Marshall stability results, where values of 10.1 kN and 6.4 kN were achieved, respectively.
- Following Table 2b asphalt mixture containing the Chornice QD filler showed the highest stiffness values, while the PKB backhouse filler and the Litice quarry dust had the lowest ones. For the QD Chornice filler and PKB backhouse filler, this finding correlates well with the Marshall stability results, where values of 14.9 kN and 7.8 kN were measured, respectively.
- In case of Table 2c the reference asphalt mix and the mix variant containing the QD Plešovice filler achieved the highest stiffness values, while the QD Litice filler had the lowest stiffness values. The remaining two variants with quarry dust as an alternative filler had stiffness values similar to the QD Litice variant. Unlike the test series with Chlum aggregate, these results are not supported by Marshall stability values, where the highest values were achieved by the QD Litice filler mix variant. Only the reference mix and the variant with the PKB backhouse filler seem to correlate reasonably well with each other.
- The stiffness values do not have a good correlation with the indirect tensile strength values either (see more in [59]). In this case, the PKB backhouse filler and the Brant backhouse filler gave the best results, followed by the QD Plešovice and QD Litice fillers. It can be concluded that the asphalt mix variants with both backhouse fillers correlate well with IT-CY stiffness and, in the case of the PKB backhouse filler, with Marshall stability as well. On the other hand, for the QD Plešovice and QD Litice fillers, the results are more consistent with the Marshall stability value.
- The IT-CY stiffness values correlate well with the Marshall stiffness results in case of asphalt mix series presented in Table 2a,b.
- Data about water resistance and the influence of the used alternative fillers on asphalt mix durability can be found e.g., in Valentin et al. [59].

### 3.2. CatBoost Modeling Results

The decision tree-based model was developed to simultaneously predict mixtures’ mechanical and volumetric properties on the basis of a few compositional variables. In particular, the inputs are represented by the main oxide contents investigated in the laboratory (SiO_2_, Al_2_O_3_, Fe_2_O_3_, CaO, and MgO), the bulk density, and a categorical variable distinguishing the three aggregate’s quarry of origin (for a total of 7 input variables). The simultaneously predicted outputs are represented by air voids content, and stiffness modulus at 15 °C.

The implemented dataset refers to the experimental investigation carried out on asphalt concretes made with 3 different aggregate types, 7 alternative fillers, and providing 6 replicates for each specimen for a total of 126 observations. The statistical description of CatBoost model variables has been provided in Table 3.

**Figure 2:**
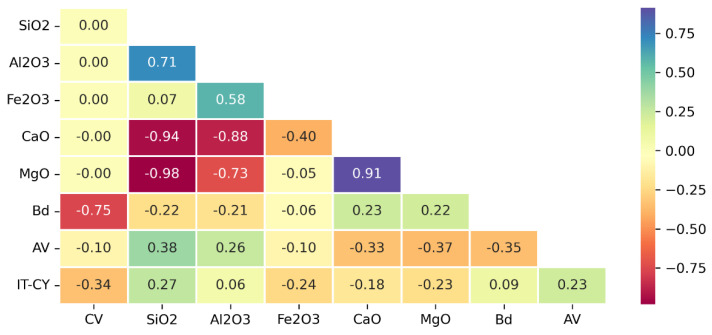
Pearson correlation matrix.

To qualitatively identify which variables are more or less correlated, the Pearson correlation matrix was realized [60]. Each element of this matrix (Figure 2) represents the strength of the correlation between variables in a pair by means of an absolute value ranging between 0 (no correlation) and 1 (perfect correlation), and a plus (direct correlation) or minus sign (inverse correlation).

By way of example, a medium positive correlation between SiO_2_ and AV [r=+0.38, n=126, p<0.0005] and a medium negative correlation between MgO and AV [r=−0.37, n=126, p<0.0005] can be observed.

**Figure 3:**
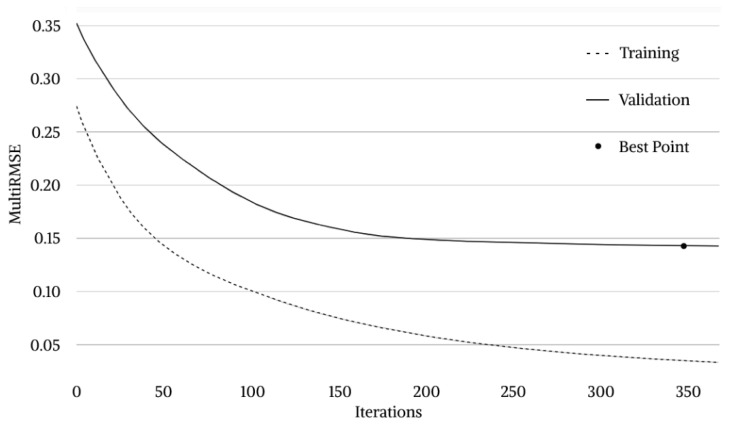
CatBoost model training process.

CatBoost model training process was represented in Figure 3. During the first 200 iterations, a significant decrease in both training and validation loss function values can be observed. During the subsequent iterations there is a continuous and gradual decrease until the best point is found and a validation MultiRMSE value of about 0.1427 is recorded. After 348th iteration, a significant decrease in the validation MultiRMSE can no longer be appreciated. Therefore, according to the overfitting detector setting, the training phase is stopped after 20 additional iterations. Best model configuration hyperparameters are then fixed so that the testing phase can begin.

To make model predictive performance more understandable, variables were denormalized and the testing results were summarized in Table 4 in terms of the six-evaluation metrics. With respect to air voids content, MAE, RMSE and R-values of about 0.20%, 0.25% and 0.97 were obtained, respectively. With respect to IT-CY, the same evaluation metrics were approximately equal to 208.50 MPa, 258.82 MPa and 0.98.

In a previous research [61], a similar database was analyzed using a model based on shallow neural networks. The neural model, on the basis of the ratios between the main oxides (always related to SiO_2_) and a categorical variable associated to the quarry/filler pair, was able to predict the average mechanical behavior of the mixtures in terms of average stiffness modulus with a coefficient of determination (R^2^) at most equal to 0.9473. In this paper, instead, the R^2^-coefficient related to the stiffness modulus was higher (equal to 0.9668) and the air voids content was predicted simultaneously with an equally high coefficient of determination (equal to 0.9229). Therefore, it could be stated that the CatBoost is roughly better than the SNN-based approach.

**Figure 4:**
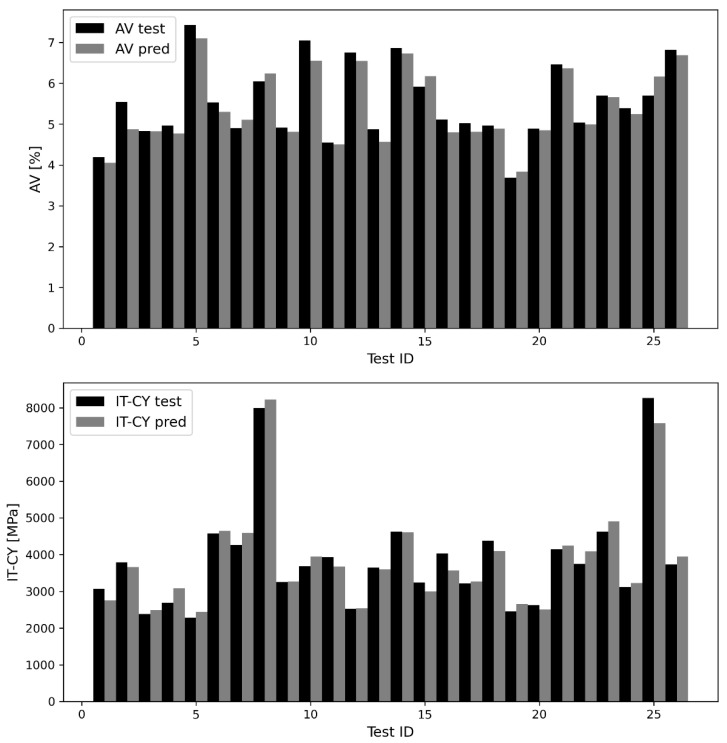
Test vectors and CatBoost-predictions of AV (**up**), and IT-CY (**down**) data.

The comparison between the test vectors and the predictions of the CatBoost model in terms of air voids content and stiffness modulus is shown in Figure 4. The black histograms stand for the experimental observations, whereas the grey ones stand for the corresponding predicted values. The ID of each AV-IT-CY test pair is represented on the horizontal axis.

It is interesting to note that, in both cases, the differences between black and gray histograms are very small. Although there are significant fluctuations in variable values, CatBoost model can follow them without ever differing too much from the corresponding true value.

**Figure 5:**
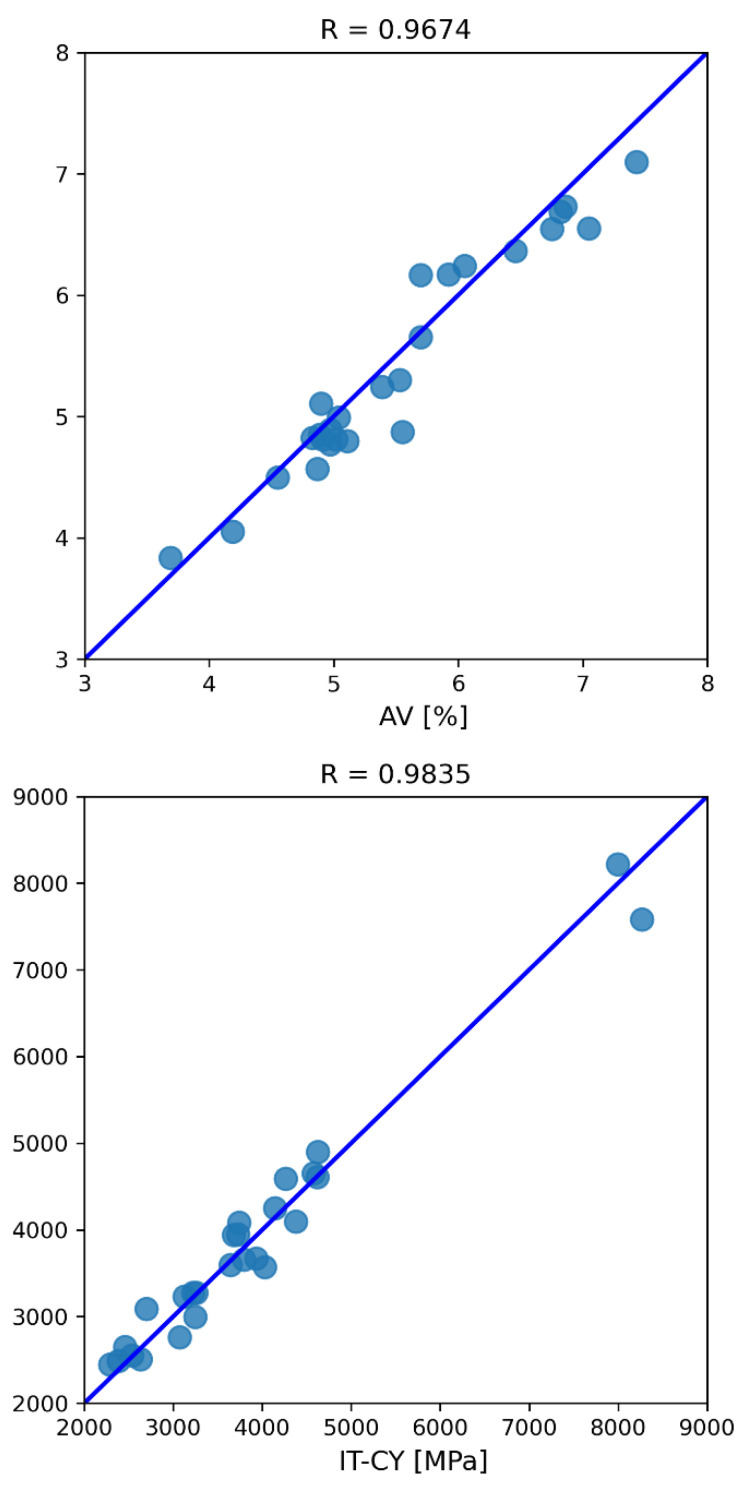
CatBoost model regression plots for AV (**up**), and IT-CY (**down**) data.

To fully appreciate prediction accuracy from a different point of view, regression plots are also shown (Figure 5). The *x*-axis represents true values, whereas the *y*-axis represents predicted ones. The line-of-equality (i.e., equivalent to 100% correspondence between observations and predictions) is represented by the blue solid line and stands for a correlation coefficient equal to 1. CatBoost predictions are represented as light blue circles and never differ too much from the line-of-equality.

Pearson correlation coefficients for air voids content and stiffness modulus resulted equal to 0.9674 and 0.9835, respectively, highlighting the remarkable performance of the model.

**Figure 6:**
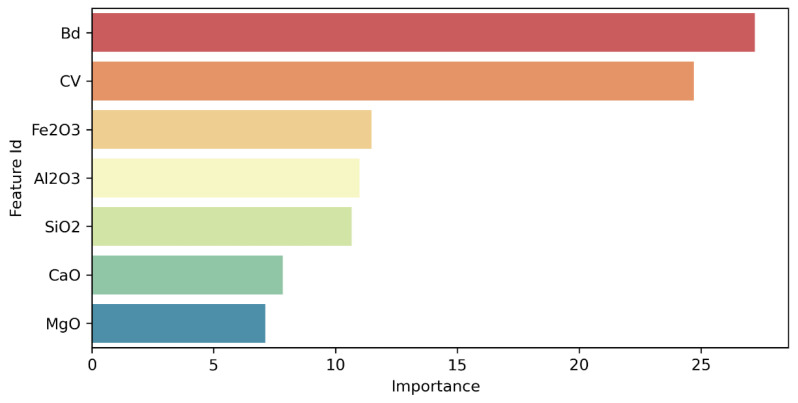
Features importance.

A sensitivity analysis was performed (Figure 6) to identify the influence each variable has on the model and its predictions. The algorithm for calculating the feature importance was implemented in Python 3.8.5, and the importance of each feature was normalized so that the sum of all the importance values was 100%. The higher the importance value, the greater the average change in the predictions if that respective feature changes. It can be observed that the bulk density has the greatest importance (27.21%), followed by the categorical variable (24.71%) and the contents of the different oxides (Fe_2_O_3_—11.47%, Al_2_O_3_—10.99%, SiO_2_—10.67%, CaO—7.84%, and MgO—7.11%).

## 4. Conclusions

The research carried out in this study fits within the context of pavement engineering and provides a useful tool for mixtures’ design that can predict their mechanical behavior on the basis of the main mineralogical composition of the filler used. A decision tree-based machine learning methodology was presented for the simultaneous prediction of mechanical and volumetric properties of asphalt concretes. An extensive laboratory investigation was carried out on 126 specimens prepared with three different quarry aggregates and with seven different quarry fillers alternative to traditional limestone and Portland cement. All the remaining compositional properties, namely aggregate grading curve, bitumen type and content, and filler content, remained essentially unchanged. X-ray fluorescence analyses were performed to determine the percentage content of five main oxides detected in the quarry fillers (SiO_2_, Al_2_O_3_, Fe_2_O_3_, CaO, and MgO). The mineralogical composition thus determined was then used as input in a CatBoost model (along with bulk density, and a categorical variable distinguishing the aggregate’s quarry of origin) in order to predict air voids content and stiffness modulus at 15 °C. The reliability of predictions was evaluated in terms of six different evaluation metrics, namely MAE, MAPE, MSE, RMSE, R, and R^2^. In particular, R^2^ values equal to 0.9229 and to 0.9668 have been obtained for air voids and stiffness modulus, thus demonstrating a good quality of predictions carried out by CatBoost algorithm. Based on the obtained results, the following conclusions can be drawn:

- The most promising results in terms of material characteristics and Marshall stability were achieved in most cases by the Chrtníky quarry dust and partially also by both tested variants of the backhouse filler. However, these fine-grained fillers did not always lead to an improvement in the stiffening effect of the mastic compared to the reference consisting of limestone filler;
- The backhouse fillers used were classified as intermediate rocks with a higher SiO_2_ content. In contrast to quarry dust, in this case, the backhouse fillers can be expected to have a finer particle size distribution, resulting in a larger specific surface area, which seems to be an important aspect, especially for achieving good resistance of the asphalt mix to the effects of water;
- The greatest stiffening effect was found for the QD Plešovice, which is considered to be an acid rock type with a high SiO_2_ content, indicating a harder parent rock compared to, for example, the Chrtníky site;
- The outlined CatBoost model allows air voids content and stiffness modulus to be accurately and simultaneously predicted;
- An XRF analysis together with simple bulk density determination could avoid the need for additional laboratory tests to experimentally determine air voids and stiffness modulus at 15 °C;

Rather than standard parameters related to the mixtures’ characterization, the main mineralogical composition was used as input of the developed model, thus representing one of the innovative aspects of this study. Furthermore, mixtures’ mechanical behavior was predicted based on an up-to-date machine learning technique, thus adding further innovation to the research.

The predictive model was developed on the basis of the experimental campaign described in this paper in which many parameters of the mixtures’ composition were kept fixed for modeling purposes. For future developments, it would be interesting to increase the size of the dataset (for example by varying filler and bitumen contents) and to include further mixture’s performance, namely fatigue life and rutting resistance.

## Figures and Tables

**Table 1:** Grid search summary.

Hyperparameter	Grid	Selected Value
Number of iterations	250, 500, 1000, 5000	500
Max depth	3, 4, 5, 6	6
Learning rate	0.1, 0.05, 0.01	0.05
k-fold Cross-validation	-	5
Overfitting detector	-	20
Loss function	-	MultiRMSE

**Table 2:** (a) Mechanical and volumetric characterization of AMs prepared with different fillers; aggregate type phonolite (Chlum quarry). (b) Mechanical and volumetric characterization of AMs prepared with different fillers; aggregate type granite porphyry (Brant quarry). (c) Mechanical and volumetric characterization of AMs prepared with different fillers; aggregate type spilite (Zbečno quarry).

**(a)**																
**Quarry**	**Filler**	**Bulk Density** **[g/cm^3^]**	**Mean**	**SD**	**Air Voids** **[%]**	**Mean**	**SD**	**Marshall Stability** **[kN]**	**Mean**	**SD**	**Marshall Flow** **[dmm]**	**Mean**	**SD**	**IT-CY @15 °C** **[MPa]**	**Mean**	**SD**
Chlum	BF Brant	2.185	2.197	0.007	6.75	6.26	0.30	6.20	5.97	0.50	2.10	2.50	0.42	2624	2961	222
		2.205			5.92			6.80			1.90			3248		
		2.193			6.42			6.10			2.50			2803		
		2.197			6.25			5.50			2.90			3067		
		2.200			6.11			5.70			2.90			2944		
		2.201			6.09			5.50			2.70			3082		
	BF PKB	2.215	2.208	0.011	6.59	6.90	0.44	6.50	5.95	0.55	2.00	2.47	0.35	2543	2483	117
		2.197			7.36			6.40			2.30			2394		
		2.195			7.43			6.40			2.20			2286		
		2.206			6.99			5.30			2.70			2552		
		2.222			6.30			5.70			2.70			2587		
		2.211			6.75			5.40			2.90			2534		
	QD Plešovice	2.296	2.306	0.016	5.95	5.54	0.66	9.90	9.40	1.71	2.30	2.57	0.26	5068	4772	924
		2.337			4.24			12.50			2.80			5104		
		2.306			5.51			7.80			2.20			2943		
		2.299			5.83			8.40			2.70			5496		
		2.295			5.98			8.30			2.60			4805		
		2.301			5.74			9.50			2.80			5216		
	QD Litice	2.346	2.345	0.003	3.74	3.80	0.12	7.90	7.75	0.48	2.50	2.75	0.29	2699	2605	193
		2.348			3.68			8.40			2.70			2852		
		2.344			3.85			8.10			2.40			2388		
		2.348			3.69			7.70			3.20			2456		
		2.344			3.87			7.20			2.80			2771		
		2.341			3.97			7.20			2.90			2464		
	Ref CaCO_3_	2.364	2.362	0.001	4.79	4.84	0.04	7.70	8.08	0.51	3.40	3.45	0.12	2527	2495	117
		2.361			4.89			8.30			3.40			2629		
		2.361			4.89			9.00			3.60			2625		
		2.362			4.86			7.60			3.30			2366		
		2.363			4.83			7.90			3.40			2383		
		2.363			4.81			8.00			3.60			2440		
	QD Chrtníky	2.365	2.361	0.003	4.72	4.88	0.13	8.70	8.42	0.28	2.90	3.10	0.18	2927	2667	205
		2.356			5.10			8.20			3.20			2299		
		2.360			4.94			8.60			3.00			2664		
		2.361			4.90			8.10			3.10			2672		
		2.364			4.77			8.70			3.40			2706		
		2.362			4.87			8.20			3.00			2734		
	QD Chornice	2.368	2.374	0.013	4.62	4.38	0.51	7.20	5.97	1.29	2.30	2.48	0.30	2636	2813	173
		2.390			3.71			7.30			2.30			2704		
		2.362			4.86			6.90			2.10			2803		
		2.385			3.92			4.70			2.70			2974		
		2.379			4.19			4.90			2.60			3069		
		2.359			4.97			4.80			2.90			2696		
**(b)**																
**Quarry**	**Filler**	**Bulk Density** **[g/cm^3^]**	**Mean**	**SD**	**Air Voids** **[%]**	**Mean**	**SD**	**Marshall Stability** **[kN]**	**Mean**	**SD**	**Marshall Flow** **[dmm]**	**Mean**	**SD**	**IT-CY @15 °C** **[MPa]**	**Mean**	**SD**
Brant	BF Brant	2.315	2.308	0.013	4.97	5.27	0.55	10.00	8.53	1.08	2.10	2.40	0.32	4581	4284	333
		2.282			6.33			9.00			2.20			3646		
		2.317			4.90			9.40			2.10			4265		
		2.313			5.04			7.50			2.90			4315		
		2.314			5.01			7.90			2.60			4415		
		2.305			5.38			7.40			2.50			4486		
	BF PKB	2.299	2.301	0.004	5.19	5.12	0.16	7.90	6.83	1.03	1.90	2.22	0.43	3366	3193	171
		2.305			4.96			7.70			1.80			3447		
		2.300			5.14			7.70			1.90			3115		
		2.294			5.39			5.80			2.40			3125		
		2.302			5.06			6.10			2.90			3083		
		2.304			4.97			5.80			2.40			3022		
	QD Plešovice	2.327	2.334	0.011	5.87	5.58	0.44	8.20	9.47	0.88	2.30	2.17	0.20	4462	4571	383
		2.330			5.74			9.80			1.90			4386		
		2.331			5.73			10.30			2.00			5300		
		2.356			4.69			10.40			2.30			4462		
		2.330			5.75			8.70			2.10			4194		
		2.331			5.70			9.40			2.40			4624		
	QD Litice	2.348	2.350	0.005	4.69	4.61	0.21	8.90	8.42	0.31	2.20	2.08	0.12	3430	3242	212
		2.349			4.65			8.50			2.10			3105		
		2.359			4.27			8.60			2.20			3565		
		2.346			4.77			8.20			2.10			3095		
		2.354			4.48			8.10			1.90			3033		
		2.345			4.82			8.20			2.00			3226		
	Ref CaCO_3_	2.360	2.369	0.008	4.90	4.53	0.34	10.00	10.02	0.81	2.50	2.70	0.28	3950	3808	172
		2.368			4.57			10.30			2.80			3763		
		2.375			4.30			9.50			2.40			3981		
		2.368			4.55			9.90			2.70			3931		
		2.361			4.87			9.00			2.60			3644		
		2.382			4.00			11.40			3.20			3578		
	QD Chrtníky	2.286	2.283	0.004	5.24	5.35	0.18	9.30	7.68	1.55	1.80	2.18	0.33	4624	4503	266
		2.276			5.63			8.50			1.80			4532		
		2.281			5.42			9.40			2.10			4726		
		2.289			5.10			6.20			2.40			4343		
		2.284			5.30			6.20			2.50			4050		
		2.281			5.42			6.50			2.50			4742		
	QD Chornice	2.354	2.328	0.014	5.14	6.01	0.52	15.80	12.60	2.58	2.60	2.92	0.42	8326	8255	440
		2.325			6.31			14.80			2.60			8204		
		2.319			6.55			14.10			2.60			7709		
		2.332			5.70			10.20			2.80			8270		
		2.323			6.05			10.40			3.40			7996		
		2.316			6.33			10.30			3.50			9025		
**(c)**																
**Quarry**	**Filler**	**Bulk Density** **[g/cm^3^]**	**Mean**	**SD**	**Air Voids** **[%]**	**Mean**	**SD**	**Marshall Stability** **[kN]**	**Mean**	**SD**	**Marshall Flow** **[dmm]**	**Mean**	**SD**	**IT-CY @15 °C** **[MPa]**	**Mean**	**SD**
Zbečno	BF Brant	2.460	2.464	0.003	5.07	4.93	0.12	7.90	6.98	1.31	25.00	30.83	5.78	3745	4071	304
		2.467			4.83			8.70			26.00			4184		
		2.461			5.04			7.80			26.00			3742		
		2.469			4.76			6.10			35.00			4424		
		2.463			4.97			5.80			38.00			4380		
		2.465			4.88			5.60			35.00			3953		
	BF PKB	2.446	2.454	0.009	6.46	6.17	0.32	8.80	7.83	1.48	25.00	29.83	4.45	4166	4354	270
		2.443			6.46			9.10			27.00			4142		
		2.448			6.46			9.60			26.00			4238		
		2.459			5.98			6.50			33.00			4300		
		2.461			5.87			6.50			32.00			4869		
		2.464			5.78			6.50			36.00			4408		
	QD Plešovice	2.408	2.451	0.045	6.86	5.32	1.60	7.40	7.37	0.39	28.00	28.67	3.98	4621	4516	162
		2.416			6.53			7.40			24.00			4425		
		2.406			6.93			7.60			24.00			4617		
		2.491			3.90			7.50			32.00			4300		
		2.492			3.85			7.70			31.00			4723		
		2.492			3.86			6.60			33.00			4408		
	QD Litice	2.512	2.509	0.002	4.78	4.88	0.09	10.10	8.70	1.25	28.00	30.00	3.41	3411	3260	91
		2.510			4.84			9.30			27.00			3279		
		2.511			4.83			10.00			26.00			3261		
		2.509			4.89			7.80			33.00			3129		
		2.505			5.02			7.30			34.00			3223		
		2.508			4.91			7.70			32.00			3257		
	Ref CaCO_3_	2.461	2.470	0.009	5.53	5.18	0.34	8.70	7.67	1.52	24.00	27.67	6.38	4576	4566	291
		2.462			5.47			9.30			21.00			4808		
		2.462			5.47			9.10			21.00			4995		
		2.475			5.00			6.50			33.00			4463		
		2.482			4.74			6.40			35.00			4328		
		2.477			4.91			6.00			32.00			4228		
	QD Chrtníky	2.486	2.485	0.014	4.82	4.85	0.52	9.40	9.17	0.62	22.00	25.83	3.25	3819	3646	273
		2.467			5.55			9.00			26.00			3791		
		2.479			5.11			9.60			22.00			4026		
		2.488			4.76			9.30			30.00			3336		
		2.484			4.88			8.00			28.00			3506		
		2.508			3.96			9.70			27.00			3399		
	QD Chornice	2.411	2.413	0.006	6.79	6.72	0.23	7.50	6.47	1.58	30.00	37.00	4.98	4168	3766	227
		2.405			7.05			7.60			34.00			3679		
		2.414			6.69			8.50			35.00			3482		
		2.422			6.38			5.30			44.00			3717		
		2.411			6.82			4.90			40.00			3729		
		2.417			6.59			5.00			39.00			3821		

**Table 3:** Statistical description of CatBoost model variables.

Variable	Description	U.M.	Count	Mean	Std Dev
CV	Categorical variable–mixture type	-	126	-	-
SiO_2_	Silicon dioxide content	[%]	126	45.17	21.10
Al_2_O_3_	Aluminum oxide content	[%]	126	16.46	5.19
Fe_2_O_3_	Ferric oxide content	[%]	126	7.43	4.83
CaO	Calcium oxide content	[%]	126	16.39	21.24
MgO	Magnesium oxide content	[%]	126	8.53	7.98
Bd	Bulk density	[g/cm^3^]	126	2.36	0.08
AV	Air Voids content	[%]	126	5.29	0.88
IT-CY	Stiffness Modulus @15 °C	[MPa]	126	3849.19	1279.28

**Table 4:** CatBoost model testing evaluation metrics.

Evaluation Metric	AV	IT-CY
MAE	0.2017	208.4975
MAPE	3.6190	5.4857
MSE	0.0645	66,987.1404
RMSE	0.2540	258.8187
R	0.9674	0.9835
R^2^	0.9229	0.9668

## Data Availability

The data that support the findings of this study are available from the corresponding author upon reasonable request.
